# Lymphocyte-to-monocyte ratio as a clinical predictor in sudden sensorineural hearing loss

**DOI:** 10.1016/j.bjorl.2025.101744

**Published:** 2025-11-26

**Authors:** Elvan Onan, Ozgur Surmelioglu, Caglar Eker, Muhammed Dagkiran, Ilda Tanrisever Pehlivan, Berk Alsancak, Suleyman Ozdemir, Ozgur Tarkan, Mustafa Mete Kiroglu

**Affiliations:** Çukurova University, Faculty of Medicine, Department of Otorhinolaryngology, Adana, Turkey

**Keywords:** Sudden Sensorineural Hearing Loss (SSNHL), Inflammatory markers, Neutrophil-to-lymphocyte ratio, Platelet-to-lymphocyte ratio, Lymphocyte-to-monocyte ratio

## Abstract

**Objective:**

This study evaluates the prognostic significance of systemic inflammatory markers ‒ NLR, LMR, PLR, and SII ‒ in patients with SSNHL.

**Methods:**

This retrospective study included 120 patients diagnosed with SSNHL at the Otolaryngology Department of Hospital. Medical records from both SSNHL and healthy control groups were reviewed to collect demographic data, pure tone audiometry results, and routine blood parameters. Hearing recovery was assessed at baseline and after two months using Siegel's criteria, and patients were categorized as either recovered (complete, partial, or slight recovery) or unrecovered.

**Results:**

Compared to controls, SSNHL patients showed significantly elevated neutrophil counts, platelet levels, NLR, PLR, and SII values. Statistically significant differences were observed between recovered and unrecovered groups in neutrophil count (p = 0.021), lymphocyte count (p = 0.001), PDW (p = 0.042), NLR (p = 0.001), PLR (p = 0.001), LMR (p = 0.001), and SII (p = 0.001). Logistic regression analysis identified low LMR as a significant independent predictor of poor prognosis (R² = 0.271).

**Conclusion:**

Elevated NLR, PLR, and SII values may reflect inflammatory processes and microvascular dysfunction in SSNHL. Among these, LMR emerged as a novel and potentially valuable prognostic biomarker for predicting treatment outcomes.

**Level of evidence:**

3.

## Highlights

• SII biomarkers are important follow-up parameters in patients with SSNHL.
• NLR, PLR, LMR, and SII can serve as vital clinical indicators for SSNHL progression.
• LMR emerges as a promising new marker for predicting patient recovery prognosis.

## Introduction

Sudden Sensorineural Hearing Loss (SSNHL) is defined as a rapid hearing loss of greater than 30 dB across three consecutive frequencies within three days, affecting many individuals annually. It is a debilitating condition characterized by a rapid decline in hearing ability, often occurring unilaterally and involving a broad spectrum of individuals globally. The exact etiology of SSNHL is not fully understood, with a range of factors, including viral infections, autoimmune mechanisms, and vascular compromise, thought to be involved in its pathogenesis.^1^ Recently, there has been growing attention on the role of inflammation in the onset and progression of SSNHL.^2^

Inflammation is a multifaceted biological response to harmful triggers, engaging immune cells and releasing inflammatory mediators. Several studies have suggested that inflammation may contribute to the pathophysiology of SSNHL by promoting cochlear damage and impairing the healing process.^3^ Chronic inflammation can directly increase the risk of ischemia by causing microvascular damage and atherogenesis. Since the cochlea’s blood supply depends on a solitary terminal artery (the labyrinthine artery) and cochlear hair cells have high oxygen demands and limited tolerance to low oxygen levels, SSNHL’s pathophysiology is believed to be closely linked to the inner ear's vascular characteristics.

Previous studies showed that the inflammatory process plays a significant role in SSNHL due to endothelial dysfunction and microvascular disorders. Systemic immune-inflammatory biomarkers like the Neutrophil-to-Lymphocyte Ratio (NLR), Lymphocyte-to-Monocyte Ratio (LMR), Platelet-to-Lymphocyte Ratio (PLR) indicate the balance of the immune response and the body's overall inflammatory status.^4^ Studies have suggested that NLR, LMR, and PLR values are correlated to an increased risk of cardiovascular mortality. Furthermore, Systemic Immune Inflammation Index (SII) is a comprehensive biomarker reflecting both overall inflammation and localized immune activity. These markers are easily calculated based on standard blood tests and are cost-effective and readily identifiable. Consequently, they have been employed in numerous clinical studies to promptly evaluate the prognostic risk associated with various diseases.

Numerous studies have evaluated the relationship between systemic inflammatory markers ‒ particularly NLR, PLR, and SII ‒ and the prognosis of sudden sensorineural hearing loss. These markers are easily accessible through routine blood tests and serve as indicators of systemic immune activation and inflammatory burden.Prior research has suggested that elevated NLR and PLR values may correlate with poorer hearing outcomes, possibly due to underlying vascular or inflammatory mechanisms affecting cochlear microcirculation.

However, while NLR and PLR have received considerable attention in this context, the LMR has been minimally studied. LMR may reflect a different dimension of the immune response, particularly balancing adaptive immunity (lymphocytes) and monocyte-driven innate inflammation. Given the emerging importance of LMR in oncology and cardiovascular diseases, its prognostic potential in SSNHL warrants further investigation.

This study aims to evaluate the prognostic significance of peripheral blood cell ratios ‒ particularly the LMR ‒ in patients with SSNHL, and to determine their association with hearing recovery. Identifying these readily accessible hematologic markers may not only aid in early risk stratification but also provide valuable insights into the underlying inflammatory mechanisms of SSNHL. By exploring their relationship with clinical outcomes, this study seeks to contribute to the development of targeted, inflammation-modulating therapeutic strategies in the management of SSNHL.

## Methods

### Study design and population

This study retrospectively analyzed 165 patients presenting with SSNHL at Çukurova University, Faculty of Medicine, Department of Otorhinolaryngology, between January 2010 and October 2023. Of these, A total of 120 patients met the inclusion criteria and were enrolled in the study. All patients were treated according to a standard protocol involving prednisone at 1 mg/kg/day, with the dose progressively reduced over at least two weeks, along with intratympanic dexamethasone injections.

The control group consisted of individuals who attended the Department of Otorhinolaryngology clinic for employment examinations, exhibited no active symptoms, and had normal pure-tone audiogram tests. Demographic data (age, sex), severity of hearing loss, blood sample results, and pure tone thresholds at the initial visit and the 2-month follow-up were extracted from medical records. The study followed the principles of the Declaration of Helsinki and obtained approval from the Institutional Review Board. (IRB Number: 142, dated Mar 8, 2024).

### Auditory evaluation

Pure tone thresholds were measured for air conduction at 250 Hz, 500 Hz, 1000 Hz, 2000 Hz, 4000 Hz, and 8000 Hz, and bone conduction at 250 Hz, 500 Hz, 1000 Hz, 2000 Hz, and 4000 Hz, respectively. Treatment response was evaluated within a maximum of 2-months based on Siegel's criteria^5^ (Table 1). Patients were then categorized into two groups: recovered (complete recovery, partial recovery, and slight recovery) and unrecovered.

**Table 1:** Siegel’s criteria of hearing improvement.

Type	Hearing recovery
I. Complete recovery	Patients whose final hearing level is better than 25 dB regardless of the size of the gain
II. Partial recovery	Patients who show more than 15 dB of gain and whose final hearing level is between 25 and 45 dB
III. Slight recovery	Patients who show more than 15 dB of gain and whose final hearing level is poorer than 45 dB
IV. No improvement	Patients who show less than 15 dB of gain

### Hematologic evaluation

Blood samples were obtained from patients at the initial presentation. The analysis of the complete blood count included WBC, neutrophil, monocyte, lymphocyte, platelet counts, MPV (Mean Platelet Volume), PDW (Platelet Distribution Width), RDW (Red Cell Distribution Width), along with NLR, LMR, PLR, and SII (total count of neutrophils × platelets divided by the lymphocyte count).

### Inclusion and exclusion criteria

Patients with confirmed SSNHL were diagnosed via pure tone audiometric tests within one week of symptom onset treated both systemic prednisone and intratympanic dexamethasone, blood samples were taken, no prior steroid treatment, and normal Magnetic Resonance Imaging (MRI) results were included in our study. Patients with systemic hypertension, diabetes mellitus, hyperlipidemia, chronic or acute renal failure, coronary artery disease, chronic liver disease, autoimmune diseases, or a history of otologic diseases such as acute and chronic otitis media and otosclerosis were excluded from the study.

### Outcome measures

The primary outcome was the association between inflammatory markers and the healing process in SSNHL patients, assessed by changes in pure-tone audiometry results at follow-up visits.

### Statistical analysis

Statistical analysis was carried out using SPSS 18.0 for Windows. Descriptive statistics were calculated, providing minimum, maximum, mean, and standard deviation for quantitative data, as well as frequency and percentage for qualitative data. Comparisons between groups were performed using the chi-square test for categorical variables, the *t*-test for continuous variables when comparing two groups, and One-Way ANOVA for comparing multiple groups, with post-hoc analysis conducted using the LSD test. A p-value < 0.05 was deemed statistically significant.

## Results

The analyses of 120 SSNHL patients and 58 control patients were examined. The mean age of the patient group was 42.96 ± 16.42, while the mean age of the control group was 38.93 ± 13.75 (p = 0.111). Fifty-five of the patients were female (54.2%), and 65 were male (45.8%). In the control group, 27 were female (52.6%), and 30 were male (47.4%). There was no statistically significant difference between the groups in terms of age and gender. When comparing the values of hemogram parameters between all patients and the control group, lymphocyte (p = 0.033) and PDW (p = 0.001) values were statistically significantly higher in the control group. At the same time, WBC (p = 0.001), neutrophil (p = 0.001), PCT (p = 0.001), MPV (0.010), NLR (p = 0.001), PLR (p = 0.001), and SII (p = 0.001) averages were statistically significantly higher in the patient group (Table 2).

**Table 2:** The demographic and clinical characteristics of the study populations.

	Patient group	Control group	p
Age	42.96 ± 16.42	38.93 ± 13.75	0.111
Gender			
Female	55 (54.2%)	27 (52.6%)	0.848
Male	65 (45.8%)	30 (47.4%)	
WBC (10^3^/μ)	9.62 ± 3.74	7.23 ± 1.83	0.001*
Neutrophil (10^3^/μ)	6.9 ± 3.5	4.21 ± 1.61	0.001*
Lymphocyte (10^3^/μ)	2.13 ± 0.91	2.46 ± 1.11	0.033*
Monocyte (10^3^/μ)	0.59 ± 0.32	0.55 ± 0.18	0.231
Platelet (10^3^/μ)	269.18 ± 72.42	250.11 ± 59.14	0.085
PCT (μg/L)	0.25 ± 0.07	0.22 ± 0.05	0.001*
MPV (fl)	9.43 ± 1.54	8.83 ± 1.15	0.010*
PDW	14.24 ± 3.13	16.75 ± 0.55	0.001*
RDW (%)	13.6 ± 1.59	13.77 ± 1.34	0.497
NLR	3.99 ± 3.17	1.93 ± 0.85	0.001*
LMR	5.76 ± 9.05	4.66 ± 2.86	0.140
PLR	150.25 ± 81.25	114.08 ± 45.06	0.001*
SII	1080.85 ± 936.92	482.86 ± 252.25	0.001*

* p < 0.05.

Patients with SSNHL were categorized into two subgroups according to whether their Pure Tone Audiometry (PTA) recovering during the follow-up periods: those who showed improvement (Siegel I-II-III) and those who did not show improvement (Siegel IV). When comparing the hematological parameters of the recovered and unrecovered groups, statistically significant differences were found in the averages of neutrophil (p = 0.21), lymphocyte (p = 0.001), PDW (p = 0.042), NLR (p = 0.001), PLR (p = 0.001), LMR (p = 0.001), and SII (p = 0.001) values. Among these parameters, neutrophil, PDW, NLR, PLR, and SII were found to be higher in the unrecovered group, while lymphocyte and LMR values were found to be higher on average in the recovered group (Table 3).

**Table 3:** The clinical characteristics of the recovered and unrecovered group.

	Recovered Group	Unrecovered Group	p
Age	4.,03 ± 15.68	43.92 ± 17.24	0.532
WBC	9.12 ± 3.47	10.13 ± 3.98	0.140
Neutrophil (10^3^/μ)	6.17 ± 3.24	7.64 ± 3.63	0.021*
Lymphocyte (10^3^/μ)	2.4 ± 0.83	1.84 ± 0.92	0.001*
Monocyte (10^3^/μ)	0.55 ± 0.25	0.65 ± 0.37	0.090
Platelet (10^3^/μ)	266.36 ± 65.96	272.08 ± 79.02	0.667
PCT (μg/L)	0.26 ± 0.08	0.25 ± 0.07	0.841
MPV (fl)	9.59 ± 1.25	9.25 ± 1.79	0.229
PDW	13.68 ± 3.12	14.83 ± 3.05	0.042*
RDW (%)	13.58 ± 1.53	13.63 ± 1.67	0.860
NLR	2.9 ± 2.1	5.11 ± 3.69	0.001*
LMR	6.94 ± 9.31	4.53 ± 8.67	0.001*
PLR	122.57 ± 45.16	178.88 ± 98.96	0.001*
SII	763.88 ± 552	1408.57 ± 1127.34	0.001*

* p < 0.05.

Furthermore, logistic regression analysis was carried out on neutrophil, lymphocyte, NLR, PLR, LMR, and SII parameters according to the recovery status of the SSNHL. The conducted modeling found that a decrease in the average values of LMR indicates a poor prognosis. (R^2^ = 0.271) (Table 4)

**Table 4:** Logistic regression analysis according to the recovery status of the patients.

	B	p	Odd’s Ratio
Neutrophil (10^3^/μ)	−0.136	0.424	0.873
Lymphocyte (10^3^/μ)	−0.006	0.990	0.994
NLR	−0.289	0.309	0.749
LMR	0.071	0.032*	1.074
PLR	−0.013	0.243	0.988
SII	0.001	0.535	1.001

* p < 0.05.

## Discussion

SSNHL is a disorder with an unknown cause, often described as idiopathic and believed to result from multiple factors. Various studies attempt to explain its cause, with inflammation, viral infection, and hypoxia being the most emphasized. Chronic inflammation can directly increase the risk of ischemia by causing microvascular damage and atherogenesis.^6^ With the cochlea's blood supply coming from a single terminal artery and its hair cells having both high oxygen needs and poor tolerance for hypoxia, the vascular status of the inner ear is believed to be a critical factor in the onset of SSNHL.^7^

Previous studies showed that the inflammatory process plays a significant role in SSNHL due to endothelial dysfunction and microvascular disorders. Systemic immune-inflammatory biomarkers like the NLR, PLR, LMR, and SII indicate the balance of the immune response and the body's overall inflammatory status. These markers are easily calculable and have been used in many studies to evaluate prognostic markers of cardiovascular diseases. Studies have shown that NLR, LMR and PLR values are associated with cardiovascular mortality.^8^

Prior studies have shown that both NLR and PLR simultaneously increase and are significant indicators of atherosclerotic disease. Additionally, levels of neutrophils and platelets reflect systemic inflammation and the extent of thrombosis.^9^ Furthermore, SII is a comprehensive biomarker reflecting both overall inflammation and localized immune activity. It is defined as the platelet count multiplied by the NLR and is a prognostic indicator for malignancy and inflammatory processes.^10^ These inflammatory markers are known to reflect both acute and chronic immune responses. In the acute phase of inflammation, such as in SSNHL, these markers can rapidly shift in response to immune activation, endothelial injury, and tissue stress. Several studies have demonstrated that these ratios fluctuate within hours to days in acute inflammatory states, including sepsis, acute coronary syndromes, and ischemic stroke. Thus, their utility in SSNHL likely reflects a similar acute-phase reaction involving vascular and immunologic mechanisms.

Neutrophil, lymphocyte and platelet count, NLR, and PLR are the most studied inflammatory biomarkers in investigating the underlying causes of SSNHL.^11^ In this study, we also evaluated LMR and SII biomarkers.

In the study by Masuda et al., elevated neutrophil counts beyond the reference range were linked to severe hearing impairment and a less favorable prognosis.^12^ Similar results were seen in the study by Koçak et al.; neutrophil counts were notably elevated in the patient group compared to the control group and were also significantly higher in the treatment-non-responsive group compared to the treatment-responsive group.^13^ Consistent with previous studies, our findings showed significantly elevated neutrophil counts in SSNHL patients ‒ particularly among those who did not recover ‒ supporting the hypothesis of neutrophil-driven inflammation in disease pathogenesis. This consistent pattern across studies reinforces the potential role of neutrophil-mediated inflammation in SSNHL pathophysiology. However, unlike previous studies, our analysis included additional markers (e.g., SII and LMR), offering a broader perspective on the systemic inflammatory response, and suggesting that while neutrophils are a key component, their predictive value may be enhanced when interpreted alongside other indices.

Kum et al. observed a significant reduction in lymphocyte counts in the patient group as opposed to the control group, with the unrecovered group showing even lower levels than the recovered group.^14^ Similar results were found in the study by Zhang et al.^15^ Our study demonstrated a significant decrease in lymphocyte counts in the patient group when contrasted with the control group, and even more pronounced reductions in the unrecovered group as compared to the recovered group. Additionally, patients with severe hearing loss had significantly lower lymphocyte values.

These consistent findings across independent studies reinforce the hypothesis that decreased lymphocyte levels may reflect an impaired immune regulatory response or excessive stress-induced lymphocyte apoptosis, both of which could contribute to poor hearing recovery. While neutrophilia indicates systemic inflammation, lymphopenia may suggest reduced immunological resilience. Therefore, monitoring lymphocyte counts could be useful not only in predicting prognosis but also in evaluating treatment response. Our data further highlight the prognostic value of lymphocyte counts, especially when considered alongside neutrophil-driven indices such as NLR or SII.

NLR serves as a marker of systemic inflammation, with its levels rising in correlation with the advancement of inflammatory conditions. Comprised of two inflammatory markers, NLR provides a more comprehensive reflection of the inflammatory state compared to neutrophils or lymphocytes alone.^16^ Similarly, PLR is recognized as a biomarker of inflammatory activity, extensively studied for its involvement in both inflammation and thrombosis,^17^ Gary et al. showed that increased PLR is important in ischemia risk.^18^ The study by Chen et al. revealed that SSNHL patients had significantly higher NLR and PLR values.^19^ Seo et al. found the same results, with NLR being an essential marker for recovery prognosis.^20^ Another study demonstrated that patients with recurrent SSNHL had significantly higher NLR and PLR values.^21^ Some studies have found the same results that NLR values affected prognosis.^13^^,^^22^ According to a meta-analysis by Niknazar et al., patients with lower PLR levels may have a more favorable prognosis.^11^ Our study supports the findings that SSNHL patients and the unrecovered group exhibit significantly higher NLR and PLR values compared to healthy individuals. However, unlike the studies mentioned above, our multivariate regression analysis did not confirm these markers as independent predictors of hearing recovery. This discrepancy may stem from methodological differences, such as variations in treatment timing, definitions of recovery, or differences in study populations and statistical approaches. Additionally, our inclusion of other systemic indices (e.g., LMR and SII) may have diluted the independent predictive strength of NLR and PLR. These findings suggest that while elevated NLR and PLR values reflect an underlying inflammatory response in SSNHL, their standalone prognostic value remains uncertain and may depend on the broader inflammatory profile.

As a prognostic indicator, SII reflects immune and inflammatory responses. Zhang et al. found SII to be an essential marker for predicting prognosis in SSNHL patients.^15^ Ulu et al. showed that a high SII value had a poor prognosis.^10^ Our study observed that SII levels were notably elevated in SSNHL patients, particularly in the unrecovered group.

Although the prognostic significance of LMR has been primarily explored in oncology and neonatal diseases, its potential value in SSNHL remains largely under-investigated. For example, previous studies have demonstrated that low LMR values are associated with poor disease-free and overall survival in breast cancer, likely reflecting elevated monocyte counts and suppressed lymphocyte-mediated immunity.^23^ Similarly, Oruz et al. found LMR to be an independent risk factor for Retinopathy of Prematurity (ROP).^24^ In the only study examining LMR in SSNHL patients, Ju et al. found that NLR, LMR, and PLR values were not significantly associated with hearing recovery in diabetic patients with SSNHL.^25^ Our findings differ from Ju et al., as we observed that a lower LMR was significantly associated with unfavorable hearing outcomes, even in a non-diabetic population. This contrast may reflect the impact of diabetes-related immunometabolic changes on LMR dynamics or suggest that LMR’s prognostic relevance may be more evident in general SSNHL populations without systemic comorbidities. Additionally, in our cohort, lower LMR values coincided with higher NLR, PLR, and SII values ‒ supporting the hypothesis that systemic inflammation and vascular injury may play central roles in SSNHL pathophysiology.

High NLR and SII values likely reflect enhanced neutrophil activity and platelet-driven vascular inflammation, while low LMR values may result from reduced lymphocyte counts due to immune suppression or stress-induced apoptosis. These shifts in leukocyte ratios may mirror the severity of systemic inflammatory response, and our findings suggest that this inflammatory burden may have a direct influence on cochlear microcirculation and treatment response. Therefore, LMR could serve as a simple yet valuable adjunct marker in the prognostic assessment of SSNHL.

Although we endeavored to conduct a comprehensive study, we must acknowledge several limitations. First, the retrospective design and brief follow-up duration are primary concerns. Second, while we included a healthy control group for baseline comparisons, we did not include a diseased control group with chronic inflammatory conditions. Such a comparison would have provided additional insight into how the studied hematologic markers behave in true pro-inflammatory states and may have strengthened the clinical context of our findings. We recognize this limitation and consider it a valuable direction for future research. Lastly, the study was limited to complete blood count analysis, with no further exploration of other inflammation markers such as C Reactive Protein (CRP). Future prospective studies with larger sample sizes and exploration at the genetic level are necessary.

## Conclusion

Our findings suggest that NLR, LMR, PLR, and SII values may be associated with hearing recovery outcomes in patients with SSNHL and could serve as accessible prognostic indicators in clinical practice. These markers can serve as vital clinical indicators for monitoring disease progression. To our knowledge, this is among the first studies to demonstrate that LMR may serve as an independent predictor of hearing recovery in SSNHL patients. Advancing research in this area will enhance the speed and accuracy of diagnosing SSNHL and foster the development of innovative treatments for patients.

## Number of authors

The number of authors is exceed seven. Our study is a study with a large number of cases. All authors contributed to the diagnosis and treatment of the patients and the writing of the article. All authors contributed to the article in various fields in the evaluation of the patient data included in the study. Our article has become more valuable with the inclusion of researchers with various experiences in the process of the existence of our article.

## ORCID ID

Elvan Onan: 0000-0003-1018-3464

Ozgur Surmelioglu: 0000-0001-5041-2802

Muhammed Dagkiran: 0000-0002-1923-3731

Ilda Tanrisever Pehlivan: 0000-0002-9134-3070

Berk Alsancak: 0009-0002-8536-2854

Suleyman Ozdemir: 0000-0002-0125-1536

Ozgur Tarkan: 0000-0002-0689-6632

Mustafa Mete Kiroglu: 0000-0002-4983-0406

## CRediT authorship contribution statement

Conceptualization: EO, MK. Data curation: EO, ITP, BA. Formal analysis: EO, CE, ITP, BA. Methodology: EO, OS, MD, SO. Project administration: EO. Supervision: MK. Writing-original draft: EO, CE, MD. Writing-review & editing: EO, OS, MD, CE, ITP, BA, SO, MK, OT. All authors read and agreed to the published version of the manuscript.

## Funding

None.

## Data availability statement

We declare that all data are available in repository.

## Declaration of competing interest

The authors declare no conflicts of interest.
